# Cost-effectiveness of using an extensively hydrolyzed casein formula plus the probiotic *Lactobacillus rhamnosus* GG compared to an extensively hydrolyzed formula alone or an amino acid formula as first-line dietary management for cow’s milk allergy in the US

**DOI:** 10.2147/CEOR.S75071

**Published:** 2015-02-27

**Authors:** Olga Ovcinnikova, Monica Panca, Julian F Guest

**Affiliations:** 1 CATALYST Health Economics Consultants, Northwood, London, UK; 2 Faculty of Life Sciences and Medicine, King’s College, London, UK

**Keywords:** amino acid formula, cost-effectiveness, cow’s milk allergy, economic evaluation, extensively hydrolyzed formula, *Lactobacillus rhamnosus* GG, Neocate, Nutramigen, US

## Abstract

**Objectives:**

The aim was to estimate the cost-effectiveness of using an extensively hydrolyzed casein formula (eHCF) plus the probiotic *Lactobacillus rhamnosus* GG (eHCF + LGG; Nutramigen LGG) compared to an eHCF alone (Nutramigen) and an amino acid formula (AAF; Neocate) as first-line dietary management for cow’s milk allergy (CMA) in the US.

**Methods:**

Using a cohort study design, the analysis was based on the case records of 136 eHCF-fed, 59 eHCF + LGG-fed, and 217 matched AAF-fed infants extracted from the Truven Health MarketScan^®^ Commercial Claims Database (a nationally representative database of the commercially insured population of the US). Clinical outcomes and health care resource use (with corresponding costs at 2012 prices), following first-line dietary management with each formula, were estimated over 12 months from the start of feeding. Differences in infants’ outcomes and resource use between groups were adjusted for any differences in baseline covariates.

**Results:**

Infants were <6 months of age at presentation. Fifty-six percent of eHCF + LGG-fed infants were estimated to have been successfully managed by 9 months compared to 38% of eHCF-fed infants and 35% of AAF-fed infants (*P*<0.05 and *P*=0.003 respectively). Infants in the AAF group used significantly more health care resources and prescribed drugs than infants in the other two groups. The estimated cost of managing a CMA infant over the first 12 months following the start of feeding was $3,577, $3,781, and $6,255 for an eHCF + LGG-fed, eHCF-fed, and AAF-fed infant, respectively. Parents’ costs accounted for up to 10% of the total costs and the remainder was incurred by insurers. The analyses were robust to plausible changes in all variables.

**Conclusion:**

Using real world evidence, initial dietary management with eHCF + LGG appears to afford a more cost-effective use of health care resources than initial dietary management with eHCF or AAF since it releases health care resources for alternative use within the system and reduces costs without impacting on the time needed to manage the allergy.

## Introduction

Cow’s milk allergy (CMA) is an abnormal immune response to milk proteins.^1^ Its incidence in infancy in Western industrialized countries has been estimated at 2%–3%,^2^,^3^ and it generally develops within the first few months of life. However, up to 90% of affected infants naturally develop tolerance to cow’s milk proteins by 5 years of age.^3^ There are several guidelines addressing the management of infants with CMA.^2^,^4^,^5^ These guidelines all recommend the use of substitutive hypoallergenic formulas,^4^,^5^ including extensively hydrolyzed formulas (eHFs) and amino acid formulas (AAFs). The clinical properties of these formulas have been reviewed elsewhere.^6^–^10^

The addition of the probiotic *Lactobacillus rhamnosus* GG (LGG) to the extensively hydrolyzed casein formula (eHCF), Nutramigen (eHCF + LGG) has been shown to accelerate the development of tolerance to cow’s milk in infants with CMA compared with those receiving other hypoallergenic formulas.^11^,^12^ In the most recent study,^12^ it was reported that significantly more infants in the eHCF + LGG group (both those with immunoglobulin E [IgE]-mediated and those with non-IgE-mediated CMA) developed tolerance to cow’s milk by 12 months (78.9%; *P*<0.05) than those fed other formulas: eHF alone (43.6%), hydrolyzed rice formulas (32.6%), soy-based formulas (23.6%), and AAF (18.2%). Binary logistic regression revealed that the rate of infants developing tolerance at the end of the study was influenced by two factors: 1) IgE-mediated mechanism (odds ratio: 0.12; *P*<0.001) and 2) the choice of formula eHCF + LGG (odds ratio: 28.62; *P*<0.001).

We have previously reported the cost-effectiveness of starting management for CMA with the eHCF, Nutramigen instead of the AAF, Neocate^13^ in the UK. This current study estimated the cost-effectiveness of using eHCF + LGG compared with eHCF alone (ie, Nutramigen) and AAF (ie, Neocate) as first-line dietary management for CMA in the US.

## Methods

### Truven Health MarketScan Commercial Claims and Encounters Database

The Truven Health MarketScan^®^ Commercial Claims Database (Truven Health Analytics Inc., Ann Arbor, MI, US) is a tool for investigating health care resource consumption in the US. The database comprises fully adjudicated and paid claims pertaining to integrated enrollment and data on inpatients, outpatients, and drugs, as well as all plan designs (ie, health maintenance organizations, preferred provider organizations, fee-for-service, etc). The data contained in the database are broadly representative of the commercially insured population of the US, with beneficiaries in all 50 states, Washington DC, Puerto Rico, and the US Virgin Islands. Data fields include, but are not limited to, descriptions of the different providers, pharmaceutical prescriptions, reimbursed clinical nutrition preparations, clinical variables (eg, symptoms, clinician visits, hospital admissions, and length of time being managed), and financial variables (ie, costs incurred by insurers and parents).

### Study population

The study population was derived from those states where eHCF + LGG, eHCF, and AAF are reimbursed. The database contained the records of 875 infants who had been diagnosed as having CMA by their pediatrician and who were managed between October 1, 2006, and October 30, 2012. Of these, 265 eHCF-fed infants were matched with 265 AAF-fed infants and 115 eHCF + LGG–fed infants were matched with 115 AAF-fed infants.

The infants fed eHCF or eHCF + LGG were <1 year of age when diagnosed with CMA, received a prescription for eHCF or eHCF + LGG as their first clinical nutrition preparation for CMA, and had at least 12 months’ follow-up data from the time of their first formula prescription. These infants were matched with AAF-fed infants according to age, sex, date of starting formula, having received a prescription for AAF as their first clinical nutrition preparation for CMA, and having at least 12 months’ follow-up data from the date of their first prescription for AAF.

129 eHCF-fed, 56 eHCF + LGG-fed, and 163 AAF-fed infants were excluded from the data set because they were premature, they had less than six prescriptions for any of the formulas of interest, or they had serious overlapping health conditions, and therefore, consumed a disproportionate amount of resource use that was not indicative of CMA. Hence, 136 eHCF-fed and 148 matched AAF-fed infants and 59 eHCF + LGG-fed and 69 matched AAF-fed infants were eligible for analysis.

The data set used for this study did not involve interaction or interview with any subjects, and the records do not include any individually identifiable data (eg, names, addresses, social security or medical record numbers, or other obvious identifiers). Consequently, this study was not research involving human subjects as defined under US law. Hence, institutional review board approval was not required.

### Study variables and statistical analyses

Information extracted from infants’ records included age and sex at baseline. Additionally, all information on CMA-related health care resource use, prescribed drug medication, prescribed clinical nutrition preparations, and costs to parents and insurers over a period of 12 months from the date of starting a formula was extracted.

Infants were assumed to have been successfully managed if they stopped using a formula and also stopped receiving medication for the symptoms of CMA, such as H_2_ antagonists, proton pump inhibitors, topical dermatologicals, and antihistamines.

Infants’ outcomes and resource use were quantified for each group. Results are presented as mean ± standard error or as percentages. Differences between groups were tested for statistical significance using a Kruskal–Wallis one-way analysis of variance or a chi-square test to determine which means or percentages were similar and which were different. These tests revealed that there were no differences between the two AAF groups across any of the studied parameters (*P*>0.820); hence they were combined to form one group of 217 patients.

Using analysis of covariance, differences in infants’ outcomes and resource use between formulas were adjusted for any differences in the following covariates: age, sex, feeding start date, and the US state in which the infants were managed. Regression analyses were used to investigate relationships between baseline variables on resource use and clinical outcomes. All statistical analyses were performed using IBM SPSS Statistics (v21.0; IBM Corporation, Armonk, NY).

### Health economic modeling

A decision model was constructed in MS Excel depicting the management of the cohort of infants in each group. The model was populated with health care resource utilization and clinical outcomes extracted from the data sets and spanned a period of 12 months from the start of feeding with eHCF + LGG, eHCF, or AAF.

#### Model outputs

The model estimated clinical outcomes and health care resource use at 12 months. Using the US inflation indexes, costs incurred by parents and insurers were uprated to 2012 prices in order to estimate the costs over 12 months from starting a formula.

#### Cost-effectiveness analyses

Effectiveness was defined as the probability of infants having been successfully managed (ie, they stopped using a formula and also stopped receiving medication for the symptoms of CMA). The cost-effectiveness of 1) eHCF + LGG compared to AAF, 2) eHCF compared to AAF, and 3) eHCF + LGG compared to eHCF was calculated as the difference between the expected management costs over 12 months from the start of feeding divided by the difference in the probability of infants being successfully managed by 12 months, and it is expressed as the cost per additional successfully managed infant. If one of the formulas improved the probability of an infant being successfully managed for less cost, it was considered to be the dominant (cost-effective) formula.

#### Sensitivity analyses

To assess uncertainty, bootstrapping was undertaken to estimate the distribution of expected costs, outcomes, and cost-effectiveness ratios. This involved generating 10,000 subsets of the data from each group on the basis of random sampling and replacing the data once sampled. Additionally, deterministic sensitivity analyses were performed on all the models’ inputs to identify how the difference in cost per infant between the groups would change by varying the value of different model inputs.

## Results

### Infants’ characteristics

Among the study population, 49% of eHCF-fed, 46% of eHCF + LGG-fed, and 52% of AAF-fed infants were female. Additionally, the mean age of the eHCF group (1.9±0.2 months) at the time of presentation was significantly lower than that of the eHCF + LGG group (2.7±0.2 months; *P*<0.05) and the AAF group (3.0±0.2 months; *P*<0.001). There was no significant difference between the mean ages of the eHCF + LGG and AAF groups.

### Infant management and outcomes

There were no significant differences in the length of time on a formula among the three groups. Ten percent of the 136 eHCF-fed infants changed to eHCF + LGG after a mean 5.4±0.3 months and 9% switched to an AAF after a mean 3.4±0.4 months, whereas 7% of the 59 eHCF + LGG-fed infants switched to an AAF after a mean 2.4±0.2 months. None of the 217 AAF-fed infants switched formulas. This is consistent with the results of our previous study^13^ in the UK, which showed that in clinical practice, 10% of eHCF-fed infants switch to an AAF and no AAF infants switch to other formulas.

Infants in the eHCF group continued feeding with a formula for a mean of 9.0±0.3 months and received a mean 8.7±0.5 prescriptions. Infants in the eHCF + LGG group continued feeding with a formula for a mean of 8.1±0.5 months and received a mean 7.9±0.4 prescriptions. Infants in the AAF group continued feeding with a formula for a mean of 9.3±0.2 months and received a mean 10.5±0.5 prescriptions per infant.

**Figure 1:**
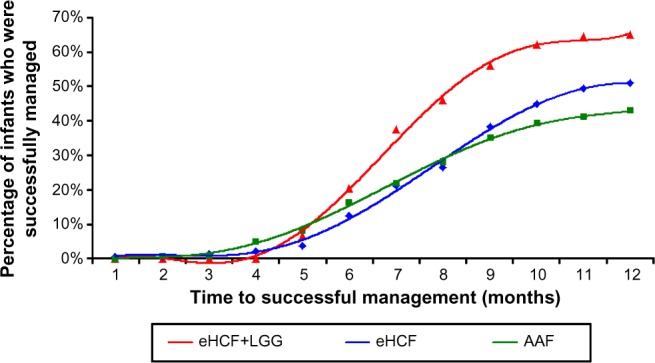
Time to successful management of cow’s milk allergy. **Abbreviations:** eHCF, extensively hydrolyzed casein formula; eHCF + LGG, extensively hydrolyzed casein formula plus the probiotic *Lactobacillus rhamnosus* GG; AAF, amino acid formula.

After 9 months from starting a formula, 56% of infants in the eHCF + LGG group were estimated to have been successfully managed because they stopped using a formula and also stopped receiving medication for symptoms of CMA. This was significantly more than the infants who were estimated to be successfully managed in both the eHCF (38%; *P*<0.05) and AAF groups (35%; *P*=0.003). There was no significant difference between the percentages of infants successfully managed in the eHCF and AAF groups at 9 months. By 12 months after having started a formula, 64% of infants in the eHCF + LGG group were estimated to have been successfully managed. This was significantly more than those estimated to have been successfully managed in the AAF group (43%; *P*=0.02). However, by 12 months, there were no longer any significant differences between the percentages of infants successfully managed in the eHCF group (51%) and the eHCF + LGG or AAF groups (Figure 1).

### Health care resource use associated with infant management

Infants in the AAF group used significantly more health care resources and prescribed drugs than infants in the other two groups (Table 1). However, there were no significant differences in hospital admissions. One infant in each group was admitted into hospital for CMA-related symptoms.

In addition to CMA-related health care resource use, four eHCF-fed infants were admitted into hospital for a mean of 6 days for asthma, pneumonia, or viral meningitis, one eHCF + LGG-fed infant was admitted into hospital for 2 days for asthma, and 15 AAF-fed infants were admitted into hospital for a mean of 3 days for asthma, bronchitis, pneumonia, gastrointestinal disease, infection, or viral meningitis during the study period. One of these infants was admitted for cardiac function tests. Additionally, in all three groups, infants received a mean of three prescriptions for an antibiotic. Infants in all three groups received <0.1 prescriptions for any other drug group.

Multiple regression analysis showed that an infant’s age at the time of starting a formula influenced the length of time on formula. The length of time on a formula decreased by 1 month for every 4 months of age (*P*=0.001).

### Health care cost of infant management

The total 12-monthly cost of infant management from starting a formula was $3,577±466 per infant in the eHCF + LGG group, $3,781±299 per infant in the eHCF group, and $6,255±225 per infant in the AAF group. Of this cost, <10% was incurred by parents (Table 2).

Use of eHCF + LGG instead of eHCF or AAF reduced the following:

1. Parent costs by $69 and $349 per infant, respectively.
2. Insurer costs by $135 and $2,330 per infant, respectively.
3. Total costs by $204 and $2,679 per infant, respectively.

### Cost-effectiveness analyses

Proportionally more infants in the eHCF + LGG group were successfully managed compared to those in the eHCF and AAF groups. Additionally, starting management with eHCF + LGG instead of eHCF or an AAF reduced costs. Hence, initial management of CMA infants with eHCF + LGG instead of an eHCF or an AAF was found to be the dominant strategy because it improved outcome for least cost. Additionally, starting management with an eHCF instead of an AAF also improved outcome for least cost. Hence, initial management of CMA infants with eHCF instead of an AAF was found to be a dominant strategy.

### Sensitivity analyses

**Figure 2:**
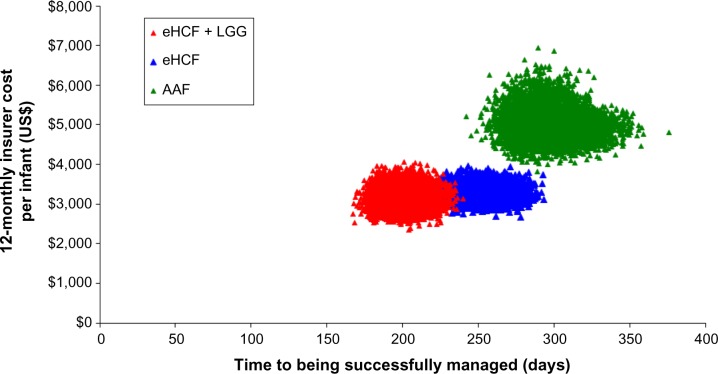
Distribution of insurers’ costs and time to being successfully managed, generated by bootstrapping. **Abbreviations:** eHCF, extensively hydrolyzed casein formula; eHCF + LGG, extensively hydrolyzed casein formula plus the probiotic *Lactobacillus rhamnosus* GG; AAF, amino acid formula.

Bootstrapping demonstrated the distribution in the insurers’ cost per infant and time to successful management. The analysis showed that the eHCF + LGG, eHCF, and AAF groups are three distinct cohorts with minimal overlap (Figure 2).

Deterministic sensitivity analyses (Table 3) showed that plausible changes in the model’s inputs did not change the finding that the cost of managing infants with eHCF + LGG was less than that of managing infants with eHCF or AAF.

## Discussion

A review of published literature suggests this to be the first study to assess the cost-effectiveness of using eHCF + LGG compared with an eHCF or AAF in the management of CMA in the US. This study made use of the complete sample of infants in the Truven Health MarketScan Commercial Claims Database who had a diagnosis of CMA and who received either first-line eHCF + LGG or eHCF and who were compared with matched infants who received first-line AAF, and who had a clinical history for at least one year. The advantage of using real world evidence from the MarketScan Commercial Claims Database is that the infant pathways and associated resource use are based on actual clinical practice rather than trial protocol-driven resource use. However, this naturalistic approach does have its limitations. Infants were not randomized to the formula they received and resource use, whilst collected prospectively, were analyzed retrospectively. There was no evidence to suggest that infants who were initially managed with an AAF had more severe symptoms. However, that possibility cannot be excluded. Undoubtedly, there would have been differences between the groups, resulting in the hospital physician’s decision to initially manage an infant with one of the formulas and the parents’ willingness to agree to feed their infant the prescribed formula. Every attempt was made to account for these differences and to overcome the nonrandomized study design. Differences in clinical outcome and resource use between formulas were adjusted for any heterogeneity in age, sex, feeding start date, and US state. Moreover, the sample sizes should have been sufficiently large enough to allow for relevant baseline differences to be apparent. Nevertheless, there will have been some differences that have not been accounted for. It is challenging to power health economic studies in which the metric is use of different resources or a range of clinical outcomes that are unknown at the outset. However, power calculations showed that the sample size was sufficiently large to detect any significant differences in resource use with 95% power and a type I (alpha) error of 0.05 between the two groups, had they occurred.

For infants to have been included in the data set for this study, their clinician had to have documented a diagnosis of CMA in their case records. However, it is unlikely that all of these infants would have undergone a double-blind placebo-controlled food challenge. Hence, it is probable that some infants did not have a differential diagnosis of CMA. As a result, infants with suspected CMA were managed by their clinician on the basis of presenting symptoms and symptom resolution. This is how many infants are diagnosed in clinical practice^13^ and therefore the diagnosis of CMA may not be secure in all cases. Therefore, we excluded those infants for whom we had some uncertainty about their diagnosis, such as those who did not have at least six consecutive prescriptions for a hypoallergenic formula. Consequently, the outcomes and estimates of health care resource use and corresponding costs in this analysis may have been derived from actual and perceived cases of CMA. Notwithstanding this, after adjusting for baseline differences, this study estimated that over the first 12 months following the start of a formula, initial use of eHCF + LGG instead of eHCF or an AAF improved outcomes and reduced both parent costs and insurer costs.

By assuming that infants had been successfully managed if they stopped using a formula and also stopped receiving medication for the symptoms of CMA, the analysis found that more infants fed with eHCF + LGG were successfully managed by 12 months than those who were fed either of the other two formulas. This trend was concordant with the findings of a study among an Italian population,^12^ which assessed the time to acquiring tolerance to cow’s milk. The Italian study evaluated tolerance acquisition in infants with IgE-mediated and non-IgE-mediated allergy separately, and tolerance development was determined by an oral food challenge.^12^ In our study, the extent of IgE involvement is unknown. Nevertheless, the Italian study previously reported that the percentage of infants developing tolerance to cow’s milk was greater among those fed eHCF + LGG than among those fed a casein-based or whey-based eHF, which was in turn greater than those fed an AAF.^12^ This trend is also consistent with the findings from other studies.^11^

There were no other published studies assessing the health economic impact of alternative formulas for the management of CMA, except our previous study,^13^ which estimated the cost-effectiveness of eHCF compared to AAF in the UK. The observations made in our UK study reinforce our current findings that eHCF affords a cost-effective use of health care resources when compared to AAF.^13^ This study is also subject to limitations that are similar to those in our UK study.^13^ The results were censored at 12 months and excluded the costs and consequences of managing infants beyond this period. The database may have underrecorded the use of some health care resources, such as some home visits made by clinicians. The analysis only considered the cost of resource use for the “average infant,” and no attempt was made to stratify resource use and costs according to sex, comorbidities, suitability of infants for different formulas, and other disease-related factors. Nevertheless, sensitivity analyses demonstrated that plausible changes in resource use had minimal effect on the relative cost-effectiveness of the three formulas. Also excluded were the indirect costs incurred by society as a result of parents taking time off work. The analysis excluded changes in quality of life and improvements in general well-being of sufferers and their parents as well as parents’ preferences. Changes in infants’ behavior were also excluded. Consequently, this study may have underestimated the relative cost-effectiveness of the formulas.

This evaluation provides an estimate of the resource implications and the associated costs and outcomes attributable to managing infants with CMA in the US, based on real world evidence. Although the study results were compelling, the analyses were based on entries in the MarketScan Commercial Claims Database and were inevitably subject to a certain amount of imprecision and lack of detail. Moreover, the computerized information in the database is collected for accounting purposes and not for research. Prescriptions issued by clinicians are recorded in the database, but it does not specify whether the prescriptions were dispensed or level of infant compliance with the product. Consequently, this study’s findings should provide the basis for a randomized controlled trial comparing the three formulas in the management of different phenotypes of CMA to prospectively measure a range of clinical outcomes and health-related quality of life, in combination with cost-effectiveness metrics.

In conclusion, within the limitations of the data set, initial dietary management with eHCF + LGG affords a more cost-effective use of health care resources than initial dietary management with eHCF or AAF because it releases health care resources for alternative use within the system and reduces costs without impacting on the time needed to manage the allergy. However, a randomized, controlled study in children receiving a probiotic-containing formula is required before this conclusion can be confirmed.

## Figures and Tables

**Table 1:** Mean cow’s milk allergy-related health care resource use per infant over the study period

	eHCF group	eHCF + LGG group	AAF group
Number of infants	136	59	217
Mean number per infant			
Outpatient visits to see a physician	2.16±1.39*	3.24±0.56*	8.88±1.05*
Diagnostic and laboratory tests	4.20±0.76**	4.68±1.18**	8.22±0.57**
Hospital admissions	0.01±0.01	0.02±0.02	0.01±0.01
Percentage of infants who received			
Gastrointestinal drugs	41%***	41%***	59%***
Topical preparations	23%	9%	15%
Antihistamines	0%	0%	1%
Adrenalin	5%	2%	9%

**Notes:** Values are shown as mean ± standard error.

* The AAF group is significantly different from the other two; *P*=0.001.

** The AAF group is significantly different from the other two; *P*=0.001.

*** The AAF group is significantly different from the other two; *P*=0.002.

**Abbreviations:** eHCF, extensively hydrolyzed casein formula; eHCF + LGG, extensively hydrolyzed casein formula plus the probiotic *Lactobacillus rhamnosus* GG; AAF, amino acid formula.

**Table 2:** Cost of infant management (in US $ at 2012 prices)

	eHCF group			eHCF + LGG group			AAF group		
	Parent cost per infant	Insurer cost per infant	Total cost per infant	Parent cost per infant	Insurer cost per infant	Total cost per infant	Parent cost per infant	Insurer cost per infant	Total cost per infant
Outpatient visits to see a physician	$72.08	$465.92	$538.00	$51.69	$420.66	$472.35	$178.30	$1,196.21	$1,374.51
eHCF	$203.14	$2,387.06	$2,590.20						
eHCF + LGG				$168.15	$2,344.55	$2,512.70			
AAF	$6.14	$227.97	$234.11	$1.69	$193.91	$195.60	$335.42	$3,853.12	$4,188.54
Other prescriptions	$66.27	$304.43	$370.70	$53.28	$285.54	$338.82	$105.28	$500.72	$606.00
Hospitalization	$0.00	$48.29	$48.29	$3.67	$53.81	$57.48	$8.35	$78.22	$86.57
Total	$347.63	$3,433.67	$3,781.30	$278.48	$3,298.47	$3,576.95	$627.35	$5,628.27	$6,255.62

**Abbreviations:** eHCF, extensively hydrolyzed casein formula; eHCF + LGG, extensively hydrolyzed casein formula plus the probiotic *Lactobacillus rhamnosus* GG; AAF, amino acid formula.

**Table 3:** Sensitivity analyses

Scenario	eHCF-fed versus eHCF + LGG-fed infants		AAF-fed versus eHCF-fed infants	
	Parents’ perspective	Insurers’ perspective	Parents’ perspective	Insurers’ perspective
The difference in the number of outpatient visits between the groups ranges from 0 to 10 visits per infant	$50–$240 per infant	$90–$500 per infant	$170–$340 per infant	$1,500–$2,600 per infant
The difference in the cash value of formulas to insurers between the groups ranges from $10 to $400 per infant		$130–$520 per infant		$2,000–$2,400 per infant
The difference in the cash value of formulas to parents between the groups ranges from $10 to $100 per infant	$70–$160 per infant		$250–$340 per infant	

**Notes:** Differences in mean costs per infant between alternative formulas from parents’ and insurers’ perspectives.

**Abbreviations:** eHCF, extensively hydrolyzed casein formula; eHCF + LGG, extensively hydrolyzed casein formula plus the probiotic *Lactobacillus rhamnosus* GG; AAF, amino acid formula.
